# Furin Prodomain ppFurin Enhances Ca^2+^ Entry Through Orai and TRPC6 Channels’ Activation in Breast Cancer Cells

**DOI:** 10.3390/cancers13071670

**Published:** 2021-04-01

**Authors:** Jose J. López, Geraldine Siegfried, Carlos Cantonero, Fabienne Soulet, Jean Descarpentrie, Tarik Smani, Iker Badiola, Simon Pernot, Serge Evrard, Juan A. Rosado, Abdel-Majid Khatib

**Affiliations:** 1 Department of Physiology, University of Extremadura, 10003 Caceres, Spain; jjlopez@unex.es (J.J.L.); carloscantonero@unex.es (C.C.); 2 LAMC, INSERM, University Bordeaux, U1029, F-33600 Pessac, France; geraldine.siegfried@inserm.fr (G.S.); fabienne.soulet@u-bordeaux.fr (F.S.); jean.descarpentrie@u-bordeaux.fr (J.D.); 3 Department of Medical Physiology and Biophysics, University of Seville, 41009 Seville, Spain; tasmani@us.es; 4 Cardiovascular Pathophysiology, Institute of Biomedicine of Seville, University Hospital of Virgen del Rocío, 41013 Seville, Spain; 5 Department of Cell Biology and Histology, Faculty of Medicine and Nursing, University of the Basque Country, 48940 Leioa, Spain; iker.badiola@ehu.eus; 6 Bergonié Institute, 33000 Bordeaux, France; s.pernot@bordeaux.unicancer.fr (S.P.); S.Evrard@bordeaux.unicancer.fr (S.E.)

**Keywords:** furin, ppFurin, breast cancer, calcium, SOCE, TRPC6, viability, migration

## Abstract

**Simple Summary:**

Furin, a proprotein convertase that belongs to a family of Ca^2+^-dependent serine peptidases, is involved in the maturation of a variety of proproteins, including growth factors, receptors and differentiation factors, adhesion molecules and proteases. Furin have been associated with tumorigenesis and tumor progression and metastasis; therefore, it has been hypothesized that Furin may constitute a new potential target for cancer therapy. In triple negative breast cancer cells, inhibition of Furin by the prodomain ppFurin results in enhancement of Ca^2+^ influx, which involves both the increase of store-operated calcium entry (SOCE) and the activation of constitutive Ca^2+^ entry. The latter involves the activation of Orai and TRPC6 channels, while the increase of SOCE observed in ppFurin-expressing cells is entirely dependent on Orai channels. As a result, ppFurin expression reduces triple negative breast cancer cell viability and ability to migrate and enhances their sensitization to hydrogen peroxide-induced apoptosis.

**Abstract:**

The intracellular calcium concentration ([Ca^2+^]_i_) modulation plays a key role in the regulation of cellular growth and survival in normal cells and failure of [Ca^2+^]_i_ homeostasis is involved in tumor initiation and progression. Here we showed that inhibition of Furin by its naturally occurring inhibitor the prodomain ppFurin in the MDA-MB-231 breast cancer cells resulted in enhanced store-operated calcium entry (SOCE) and reduced the cell malignant phenotype. Expression of ppFurin in a stable manner in MDA-MB-231 and the melanoma MDA-MB-435 cell lines inhibits Furin activity as assessed by in vitro digestion assays. Accordingly, cell transfection experiments revealed that the ppFurin-expressing cells are unable to adequately process the proprotein convertase (PC) substrates vascular endothelial growth factor C (proVEGF-C) and insulin-like growth factor-1 receptor (proIGF-1R). Compared to MDA-MB-435 cells, expression of ppFurin in MDA-MB-231 and BT20 cells significantly enhanced SOCE and induced constitutive Ca^2+^ entry. The enhanced SOCE is impaired by inhibition of Orai channels while the constitutive Ca^2+^ entry is attenuated by silencing or inhibition of TRPC6 or inhibition of Orai channels. Analysis of TRPC6 activation revealed its upregulated tyrosine phosphorylation in ppFurin-expressing MDA-MB-231 cells. In addition, while ppFurin had no effect on MDA-MB-435 cell viability, in MDA-MB-231 cells ppFurin expression reduced their viability and ability to migrate and enhanced their sensitization to the apoptosis inducer hydrogen peroxide and similar results were observed in BT20 cells. These findings suggest that Furin inhibition by ppFurin may be a useful strategy to interfere with Ca^2+^ mobilization, leading to breast cancer cells’ malignant phenotype repression and reduction of their resistance to treatments.

## 1. Introduction

Breast cancer is one of the most commonly diagnosed cancers in women, and the incidence of this cancer is continuously increasing. Even though treatment of breast cancer has improved in recent years, more than 600,000 people still die from this disease every year [1]. Like various cancers, breast cancer directly involves changes in intracellular Ca^2+^ dynamics [2,3]. Such variations are frequently associated with intensification of cancer-related processes, including cell proliferation, migration, and/or escaping of cell apoptosis [2,3]. In breast cancer cells, the levels of Ca^2+^ can be increased by the influx of extracellular Ca^2+^ via the Ca^2+^ channels in the plasma membrane. Among the mechanisms for Ca^2+^ influx, store-operated Ca^2+^ entry (SOCE), a mechanism triggered by the depletion of the intracellular Ca^2+^ stores, plays a major role in cell biology. SOCE has been shown to be required for the development of most cancer hallmarks including proliferation, migration, angiogenesis, and apoptosis resistance [4,5]. The key components of SOCE are Stromal interaction molecule 1 (STIM1), the sensor of endoplasmic reticulum Ca^2+^ levels, and the Ca^2+^-permeable channels of the Orai family in the plasma membrane [6]. The remodeling of Ca^2+^ homeostasis can be mediated by changes in the expression of these channels and Ca^2+^ pumps as well [7]. Thus, in triple negative breast cancer cells, such as MDA-MB-231 cells, SOCE has been reported to be strongly dependent on STIM1 and Orai1 [8], with TRPC6 playing a relevant role in the surface expression of Orai1 [9], while in luminal breast cancer cells SOCE is mostly mediated by STIM1, STIM2, and Orai3 [8]. In many circumstances, changes in [Ca^2+^]_i_ are controlled by intracellular Ca^2+^ store mobilization and Ca^2+^ influx through hormones and growth factor receptor activation [2,3,7]. Interestingly, a wide range of these secretory proteins are synthesized as inactive precursors that are activated following their cleavage by Furin-like enzymes at the general motif (K/R)-(X)n-(K/R)↓, where X is any amino acid and *n* = 0, 2, 4, or 6 [10,11,12,13].

Furin, also known as proprotein convertase subtilisin/kexin 3 (PCSK3), is a proprotein convertase that belongs to a family of Ca^2+^-dependent serine peptidases. Synthesized as a precursor protein (preppFurin), this enzyme requires autocleavage in the secretory pathway for its activation [10,14,15]. Following the removal of the “pre” signal peptide in the endoplasmic reticulum (ER), the remaining protein is then autocatalytically processed in the secretory pathway to generate the N-terminal ppFurin fragment and mature active Furin. N-terminal ppFurin fragment is associated with the enzyme functions as a potent inhibitor [10,14,15]. The presence of a C-terminal membrane binding domain [10,14,15] in the mature Furin allows this enzyme to cycle between the Golgi and plasma membrane that mediates the maturation of a strikingly varied set of proproteins. These include growth factors, receptors and differentiation factors, adhesion molecules, and proteases, which have been associated with tumor initiation and different stages of tumor progression, angiogenesis, immune response, and metastasis [10,11,16,17,18,19]. Based on various studies describing the activation of many proteins implicated in neoplasia by Furin, such as growth factors, their receptors, proteases, and adhesion molecules, it was hypothesized that this protease may constitute a new potential target for cancer therapy [10,11,16,17,18]. Subsequently, comparative analysis of normal and tumor tissues derived from patients revealed altered expression levels of Furin in various types of cancers including breast cancer [10,20].

To date, much of the research exploring calcium signaling in cancer has focused on studying changes in the expression levels of proteins directly involved in the regulation of cellular Ca^2 +^ mobilization including calcium channels and pumps. Using gene silencing approaches and/or chemical inhibitors and activators, the role of these proteins in calcium signaling and the malignant phenotype of cancer cells has been previously evaluated. However, little is known about the regulation of the expression and/or activity of these calcium regulator proteins by endogenous cellular processes that occur in cancer cells, particularly enzymes such as Furin that mediate the activation of various protein precursors involved in neoplasia through calcium homeostasis alteration. Revealing such information would advance our understanding of the mechanisms essential for cancer progression, and may allow the identification of potential therapeutic targets involved in the control of calcium regulators expression. In this study, we aimed to characterize Ca^2+^ changes in response to Furin repression by ppFurin in breast cancer cells, with a focus on investigating the relationship between Ca^2+^ concentration changes, ion channels’ activation, and malignant phenotype repression by ppFurin. Our results indicate that ppFurin enhances Ca^2+^ influx, leading to attenuation of breast cancer cell viability and migration and enhanced sensitization to the apoptotic agent hydrogen peroxide. These studies provide new insights into the consequences of Furin activity on Ca^2+^ homeostasis in breast cancer cells.

## 2. Results

### 2.1. Furin Activity Is Repressed by ppFurin in Breast Cancer Cells

**Figure 1:**
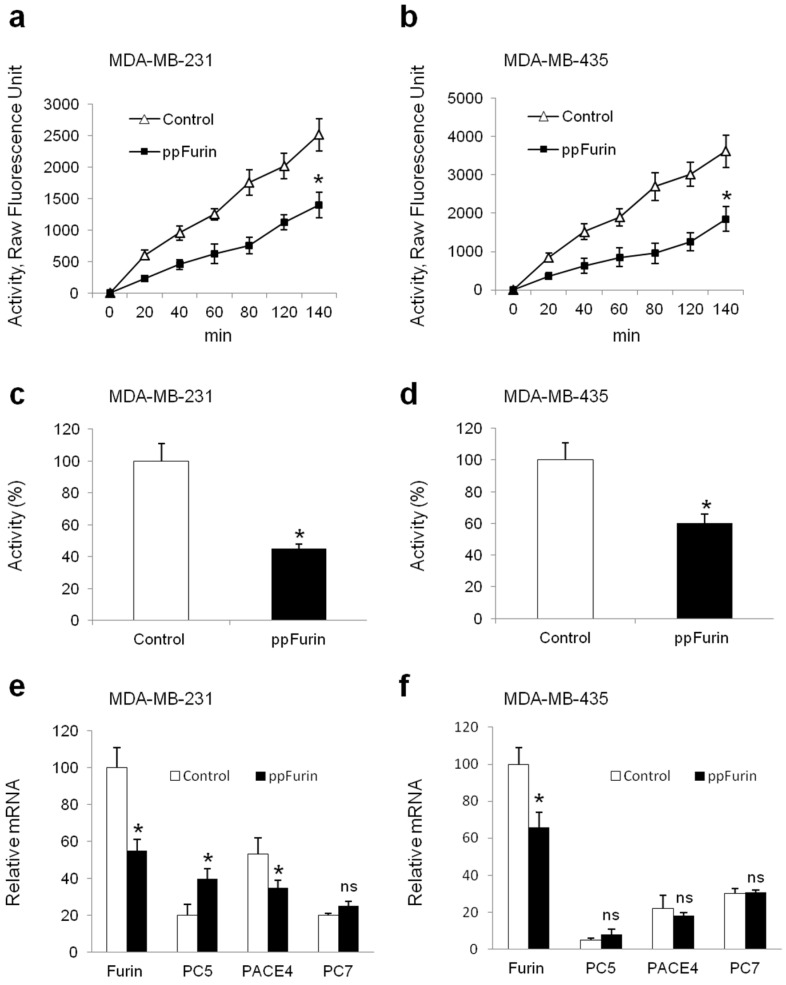
Inhibition of Furin activity by ppFurin in breast MDA-MB-231 and melanoma MDA-MB-435 cell lines. (**a**,**b**) MDA-MB-231 and MDA-MB-435 cells were stably transfected with pIRES2-EGFP empty vector (control) or pIRES2-EGFP-ppFurin and the proprotein convertase (PC) activity was evaluated by the measurement of cell lysates’ ability to digest the universal PC substrate pERTKR-MCA at the indicated time periods. (c,d) Bar graphs representing PCs activity of MDA-MB-231 and MDA-MB-435 cells at 2 h of incubation in the absence and presence of ppFurin. (**e**,**f**) Real-time PCR analysis of indicated PC expression in control and ppFurin-expressing cells. Results are shown in the bar graphs and are expressed as the percentage of enzymatic activity or transcripts relative to controls (100%). All values are presented as means ± SEM of three independent experiments. Statistical significance was assessed by *t*-test and * represents *p* < 0.05 as compared to the corresponding controls.

To investigate the role of Furin in the regulation of Ca^2+^ mobilization in breast cancer cells, MDA-MB-231 were stably transfected with pIRES2-EGFP empty vector (control) or containing ppFurin cDNA. For comparison, the melanoma cells MDA-MB-435 were also transfected with pIRES2-EGFP empty vector (control) or containing ppFurin. Using an in vitro enzymatic digestion assay [21,22] and the universal proprotein convertase (PC) substrate, the fluorogenic peptide pERTKR-MCA, revealed that while high PC activity was detected in control MDA-MB-231 cells, the expression of ppFurin in these cells inhibited PCs enzymatic activity (Figure 1a,c). Similarly, the expression of ppFurin in the melanoma cell line MDA-MB-435 also represses the PC activity (Figure 1b,d). Using specific primers for the PCs found in the secretory pathway, namely Furin, PC5, PAEC4, and PC7 real-time PCR confirmed the presence of all these PCs in control MDA-MB-231 and MDA-MB-435 cells, while the expression of ppFurin in MDA-MB-231 repressed the mRNA level of Furin and PACE4 and had no effect on PC7 enhanced PC5 expression, as previously reported [14]. In MDA-MB-435 the expression of ppFurin only repressed the expression of Furin and had no effect on the analyzed PCs (Figure 1e,f).

**Figure 2:**
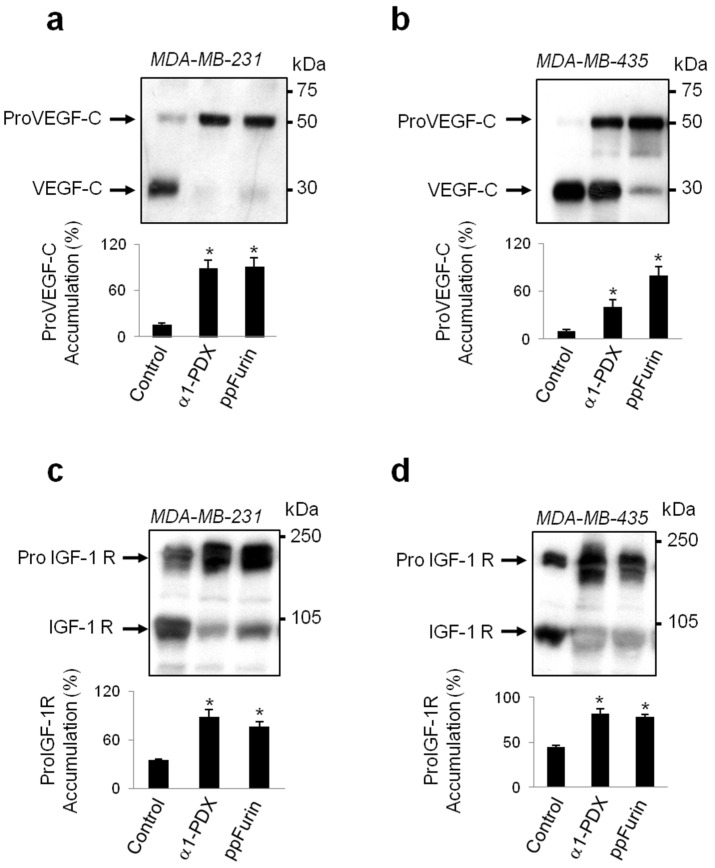
Inhibition of vascular endothelial growth factor C (proVEGF-C) and insulin-like growth factor-1 receptor (proIGF-1R) by ppFurin in breast MDA-MB-231 and melanoma MDA-MB-435 cell lines. Western blotting for proVEGF-C precursor and its mature form (**a**,**b**) and for proIGF-1R precursor and its mature form (**c**,**d**) expression. Effect of the general PC-inhibitor α1-antitrypsin Portland (α1-PDX) on proVEGF-C and proIGF-1R cleavage is given for comparison. Bar graphs indicate the percentages of proVEGF-C and proIGF-1R accumulations in the absence and presence of ppFurin or α1-PDX. All values are presented as means ± SEM of three independent experiments. Statistical significance was assessed by *t*-test and * represents *p* < 0.05 as compared to the corresponding controls.

To further confirm the effect of ppFurin on the enzymatic activity in MDA-MB-231 and MDA-MB-435 ppFurin-expressing cells, we next assessed in these cells their ability to convert the human proVEGF-C into the mature VEGF-C form, previously reported to be processed by Furin and other PCs [23]. Immunoblotting analysis revealed that media derived of MDA-MB-231 and MDA-MB-435 cells transfected with a vector encoding proVEGF-C mostly express the mature form of VEGF-C. Transfection of ppFurin expressing cells with a vector encoding proVEGF-C showed reduction of the mature protein and enhanced the accumulation of the unprocessed form of VEGF-C (Figure 2a,b). Similarly, immunoblotting analysis of the processing of the PC substrate insulin-like growth factor-1 receptor (proIGF-1R) revealed that MDA-MB-231 and MDA-MB-435 cell lines express proIGF-1R of which the processing of the β-subunit was also blocked in MDA-MB-231 and MDA-MB-435 cells in the presence of ppFurin, as evidenced by the accumulation of the precursor form and the reduction of the mature form of IGF-1R. For comparison the processing of proVEGF-C and proIGF-1R was also examined in control cells and in the presence of the general PC-inhibitor (α1-antitrypsin Portland (α1-PDX). As illustrated, in α1-PDX-expressing cells, the accumulation of proVEGF-C and proIGF-1R precursor forms was also enhanced (Figure 2). Compared to MDA-MB-231 cells, α1-PDX failed to completely block the processing of proVEGF-C in MDA-MB-435 (Figure 2a,b), suggesting a presence in these cells’ high PCs levels and/or activity involved in proVEGF-C processing that is not totally repressed by this inhibitor.

### 2.2. Regulation of Ca^2+^ Mobilization by ppFurin

**Figure 3:**
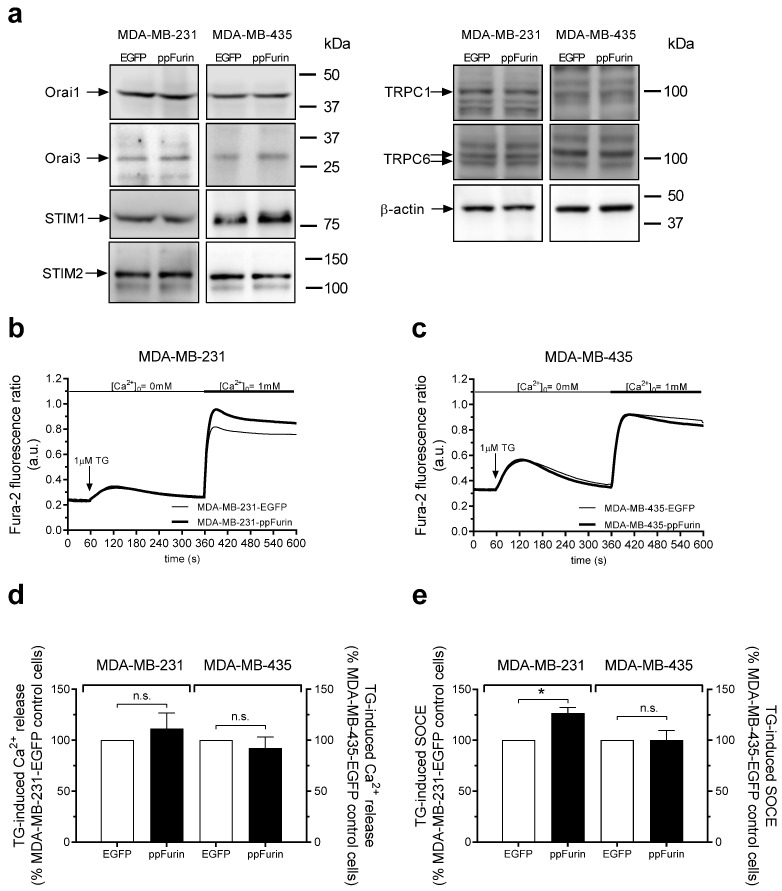
ppFurin enhances store-operated Ca^2+^ entry (SOCE) in MDA-MB-231 cells. (**a**) Control MDA-MB-231 and MDA-MB-435 cells or expressing ppFurin were lysed and whole cell lysates were analyzed by Western blotting using anti-Orai1, Orai3, STIM1, STIM2, TRPC1, TRPC6, or β-actin antibody, as indicated. Blot images are from one experiment representative of three which gave similar results. (**b**–**e**). Changes in [Ca^2+^]_I_ were detected in fura-2-loaded control MDA-MB-231 and MDA-MB-435 cells or expressing ppFurin. Cells were stimulated with 1 µM of TG in a Ca^2+^-free medium (100 µM of EGTA added), and later SOCE was detected by the addition of 1 mM of CaCl_2_ to the extracellular medium. (**b**,**c**) Traces are representative of 40 cells/day/3–5 days. (**d**,**e**) Histograms indicate TG-induced Ca^2+^ release and SOCE as the area under the curve, expressed as mean ± SEM and presented as percentage of their respective control cells. Statistical significance was assessed by Student’s *t*-test and * represents *p* < 0.05 as compared to cells transfected with pIRES2-EGFP empty vector.

Next, we investigated the possible effect of ppFurin on Ca^2+^ mobilization in MDA-MB-231 cells. First, we analyzed the effect of overexpression of ppFurin on the expression level of several key elements of Ca^2+^ influx—Orai1, Orai3, STIM1, STIM2, TRPC1, and TRPC6 in MDA-MB-231 and MDA-MB-435 cells—and our results indicate that expression of ppFurin does not significantly alter the expression of these proteins in these cells (the expression of Orai1, Orai3, STIM1, STIM2, TRPC1, and TRPC6 was 101 ± 9%, 110 ± 15%, 103 ± 8%, 98 ± 8%, 98 ± 18%, and 94 ± 10% of control, respectively, in ppFurin-expressing MDA-MB-231 cells, and 98 ± 8%, 104 ± 10%, 102 ± 13%, 94 ± 10%, 102 ± 11%, and 106 ± 10% of the control, respectively, in ppFurin-expressing MDA-MB-435 cells; Figure 3a; *n* = 4). As depicted in Figure 3b, in cells stably transfected with pIRES2-EGFP empty vector perfused with a Ca^2+^-free medium, treatment with the SERCA inhibitor Thapsigargin (TG) led to a transient increase in [Ca^2+^]i as a consequence of Ca^2+^ release from the intracellular stores. Subsequent re-addition of CaCl_2_ to the extracellular medium resulted in a further increase in [Ca^2+^]i indicative of SOCE. Expression of ppFurin significantly enhanced TG-evoked SOCE without having any effect on Ca^2+^ release from the intracellular stores (Figure 3b,d,e). The effect of ppFurin was specific for MDA-MB-231 cells as it did not have any effect on TG-induced Ca^2+^ mobilization in the melanoma cell line MDA-MB-435 (Figure 3c–e).

**Figure 4:**
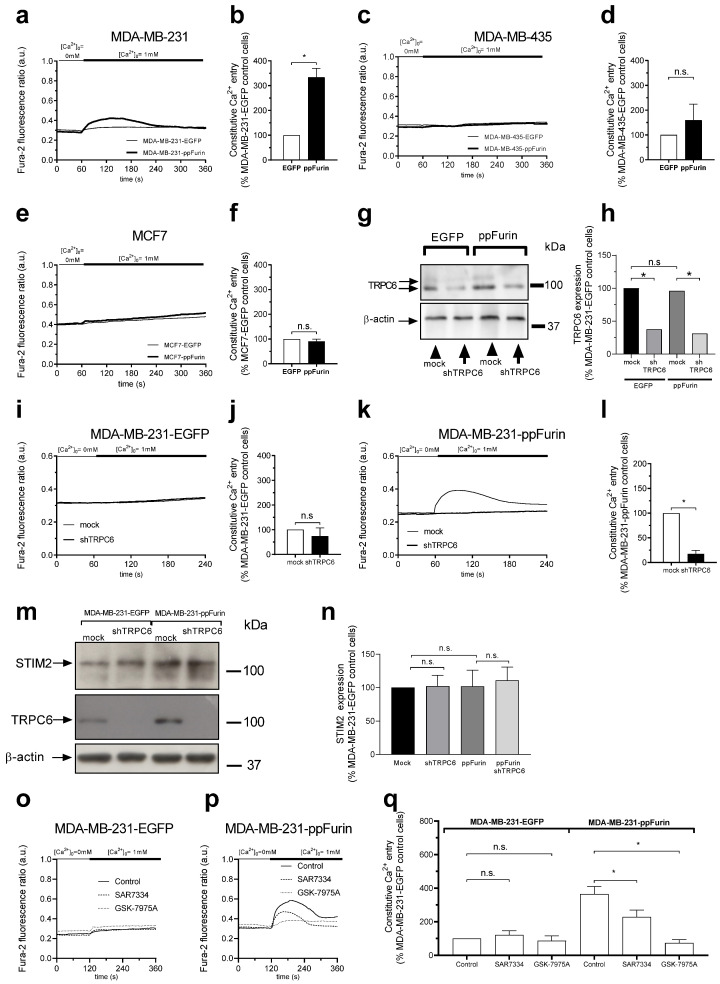
ppFurin induces constitutive Ca^2+^ entry in MDA-MB-231 cells. (**a**–**f**) Changes in [Ca^2+^]_I_ were detected in fura-2-loaded control MDA-MB-231 and MDA-MB-435 and MCF7 cells or expressing ppFurin. Cells were maintained in a Ca^2+^-free medium (100 µM of EGTA added), and later 1 mM of CaCl_2_ was added in the extracellular medium to detect the existence of a constitutive Ca^2+^ entry. Each trace is representative of 40 cells/day/3–5 days. Histograms represent constitutive Ca^2+^ entry as the area under the curve, expressed as mean ± SEM and presented as the percentage of their respective control cells. Statistical significance was assessed by Student’s *t*-test and * represents *p* < 0.05 as compared to the corresponding control. (**g**,**h**) Control and ppFurin-expressing MDA-MB-231 cells were transfected with shTRPC6 or scramble plasmid, as indicated. TRPC6 and β-actin detection was carried out at 48 h post-transfection by Western blotting as described in the Material and Methods section. Blot image is from one experiment representative of five which gave similar results. Histograms show TRPC6 expression represented as percentage of control and expressed as mean ± SEM. Statistical significance was assessed by one-way ANOVA combined with post-hoc Tukey’s test. * *p* < 0.05 as compared to the corresponding control. (**i**–**l**) Control and ppFurin-expressing MDA-MB-231 and MDA-MB-435 cells were transfected with shTRPC6 or scramble plasmid, as indicated. Loading cells with Fura-2 was carried out at 48 h after transfection. Cells were maintained in a Ca^2+^-free medium (100 µM of EGTA added), and later 1 mM of CaCl_2_ was added in the extracellular medium to detect the existence of a constitutive Ca^2+^ entry. Traces are representative of 40 cells/day/3–5 days. Histograms represent constitutive Ca^2+^ entry as the area under the curve, expressed as mean ± SEM and presented as the percentage of their respective control cells. Statistical significance was assessed by Student’s *t*-test and * represents *p* < 0.05 as compared to the corresponding control. (**m**,**n**) Control and ppFurin-expressing MDA-MB-231 cells were transfected with shTRPC6 or scramble plasmid, as indicated. TRPC6, STIM2, and β-actin detection was carried out at 48 h post-transfection by Western blotting as described in the Material and Methods section. Blot image is from one experiment representative of five which gave similar results. Histograms represent STIM2 expression represented as percentage of Control and expressed as mean ± SEM. Statistical significance was assessed by one-way ANOVA (n = 5). (**o**–**q**) Fura-2-loaded control and ppFurin-expressing MDA-MB-231 cells were pretreated for 10 min with 1 µM of SAR7334 or for 1 min with 10 µM of GSK-7975A in a Ca^2+^-free medium (100 µM of EGTA added) and then 1 mM of Ca^2+^ was added to estimate constitutive Ca^2+^ entry. Traces are representative of 40 cells/day/3–5 days. Bar graphs represent constitutive Ca^2+^ entry as the area under the curve, expressed as mean ± SEM and presented as the percentage of control MDA-MB-231-EGFP cells. Statistical significance was assessed by one-way ANOVA combined with post-hoc Tukey’s test. * *p* < 0.05 as compared to the corresponding control.

Furthermore, we found that expression of ppFurin in MDA-MB-231 cells leads to transient constitutive Ca^2+^ entry detected as a rise in [Ca^2+^]_i_ upon re-addition of extracellular Ca^2+^ in the absence of stimuli, while this effect was absent in MDA-MB-231, MDA-MB-435, and MCF7 control cells and MDA-MB-435 and MCF7 cells expressing ppFurin (Figure 4a–f). In MDA-MB-231 cells, both shRNA- and CRISPR/Cas9-based assays revealed that SOCE is mediated by STIM1 and Orai1 [8,24]. In addition, we found that TRPC6 is upregulated in MDA-MB-231 cells and plays a relevant role in the activation of SOCE in these cells [9]. Hence, we have investigated whether TRPC6 mediates the increase in SOCE as well as the constitutive Ca^2+^ entry observed in MDA-MB-231 cells in the presence of ppFurin. As depicted in Figure 4g–l, TRPC6 expression silencing inhibits constitutive Ca^2+^ entry in ppFurin-expressing cells, without having any effect on cells expressing empty vector. We further explored the role of TRPC6 in constitutive Ca^2+^ entry in ppFurin-expressing cells by pharmacological inhibition of the channel using SAR7334. This agent is a TRPC6 inhibitor that might also have some effect on TRPC3 and TRPC7 [25], although the expression of the latter has not been detected in MDA-MB-231 cells [26]. Treatment of ppFurin-expressing and control MDA-MB-231 cells for 10 min with 1 µM of SAR7334 significantly attenuated constitutive Ca^2+^ entry in ppFurin-expressing cells (Figure 4o–q). Altogether, these findings indicate that TRPC6 is involved in constitutive Ca^2+^ entry in ppFurin-expressing MDA-MB-231 cells. Recent studies have reported constitutive activity of STIM2 in resting conditions [27,28]. Hence, we have investigated whether STIM2 expression is altered in ppFurin-expressing cells or upon TRPC6 expression silencing. However, our results indicate that neither ppFurin expression nor TRPC6 expression silencing significantly alter STIM2 expression (Figure 4m,n); nonetheless, the role of STIM2 on ppFurin-induced Ca^2+^ entry cannot be excluded. Finally, we further investigated the role of Orai channels in constitutive Ca^2+^ entry in ppFurin-expressing cells. Treatment of ppFurin-expressing and control MDA-MB-231 cells for 1 min with 10 µM of GSK-7975A significantly reduced constitutive Ca^2+^ entry in ppFurin-expressing MDA-MB-231 cells, without having any effect on control cells (Figure 4o–q). As GSK-7975A is able to block both Orai1 and Orai3 at the concentration used [29], these findings suggest that Orai channels might also be involved in the constitutive Ca^2+^ entry in ppFurin-expressing MDA-MB-231 cells.

**Figure 5:**
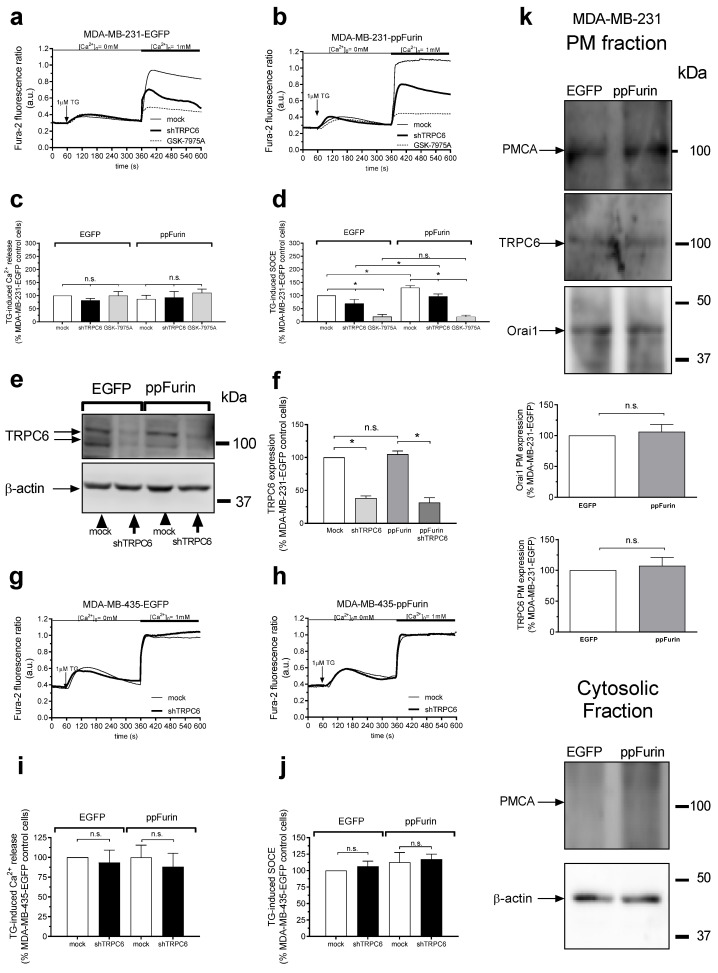
Orai channels play a relevant role in the enhanced SOCE induced by ppFurin in MDA-MB-231 cells. Control (**a**,**c**) and ppFurin-expressing MDA-MB-231 cells (**b**,**d**) were either transfected with shTRPC6 or scramble plasmid or pretreated for 1 min with 10 µM GSK-7975A. Later, cells were loaded with Fura-2 and perfused with a Ca^2+^-free medium (100 µM of EGTA added). Ca^2+^ store depletion was triggered by the stimulation of the cells with 1 µM of TG, and later 1 mM of Ca^2+^ was added to the extracellular medium to initiate SOCE. Traces are representative of 40 cells/day/3–5 days. Bar graphs indicate TG-evoked Ca^2+^ release and Ca^2+^ entry, as indicated, as the area under the curve, expressed as the mean ± SEM and presented as the percentage of the control (MDA-MB-231-EGFP control cells). Statistical significance was assessed by Student’s *t*-test and * represents *p* < 0.05 as compared to the corresponding control. (**e**,**f**) Control and ppFurin-expressing MDA-MB-231 cells were transfected with shTRPC6 or scramble plasmid and 48 h later were lysed. TRPC6 and β-actin expression were determined by Western blotting as described in the Material and Methods section. Bar graphs represent TRPC6 expression under the different experimental conditions. Data are expressed as mean ± SEM. Statistical significance was assessed by Student’s *t*-test and * represents *p* < 0.05 as compared to the corresponding control. Control (**g**,**i**) and ppFurin-expressing MDA-MB-435 cells (**h**,**j**) were transfected with shTRPC6 or scramble plasmid. Fura-2-loaded cells were perfused with a Ca^2+^-free medium (100 µM of EGTA added) and then were stimulated with 1 µM of TG, and later SOCE was detected by re-addition of 1 mM of Ca^2+^ to the extracellular medium. Traces are representative of 40 cells/day/3–5 days. Bar graphs represent TG-evoked Ca^2+^ release and Ca^2+^ entry, as indicated, as the area under the curve, expressed as mean ± SEM and presented as percentage of control (MDA-MB-435-EGFP control cells). Statistical significance was assessed by Student’s *t*-test. (**k**) MDA-MB-231 cells plasma membrane resident proteins were labeled by biotinylation, as described under the Material and Methods. TRPC6 and Orai1 localization in the plasma membrane (PM)fraction were detected by Western blotting using anti-TRPC6 or anti-Orai1 antibody, respectively. The expression of plasma membrane Ca^2+^-ATPase (PMCA) in both fractions, PM and cytosolic fractions, was also detected using the anti-PMCA antibody, as the control. These results are representative of four separate experiments. Bar graphs represent the quantification of Orai1 and TRPC6 surface exposition. Data are expressed as mean ± SEM. Statistical significance was assessed by Student’s *t*-test.

We further explored the mechanism underlying the enhanced SOCE in cells expressing ppFurin. As depicted in Figure 5a–d, our results indicate that treatment of control and ppFurin-expressing MDA-MB-231 cells with the Orai channel inhibitor GSK-7975A significantly inhibited SOCE without having any effect on Ca^2+^ release from the intracellular stores. Interestingly, the remnant SOCE after treatment with GSK-7975A was similar in control and ppFurin-expressing cells, thus suggesting that Orai channels play a relevant role in the enhanced SOCE observed in ppFurin-expressing cells. As previously reported [9], attenuation of TRPC6 expression in MDA-MB-231 cells transfected with pIRES2-EGFP empty vector or ppFurin significantly inhibits SOCE (Figure 5a–d), and the inhibition of SOCE upon TRPC6 knockdown was comparable in control and ppFurin-expressing cells (Figure 5a,b,d). As a result, after TRPC6 knockdown, SOCE in ppFurin-expressing cells was 138% of control (Figure 5d; *p* < 0.05), thus suggesting that TRPC6 has no significant role in the enhanced SOCE observed in cells expressing ppFurin. TRPC6 silencing in MDA-MB-435 cells was without effect on TG-evoked Ca^2+^ release and entry either in control or ppFurin-expressing cells (Figure 5g–j).

We further assessed whether the mechanism underlying the enhanced SOCE in ppFurin-expressing cells is associated with a greater expression of Orai1 channels. Hence, Orai1 plasma membrane expression was assessed by biotinylation in control and ppFurin-expressing MDA-MB-231 cells. As shown in Figure 5k, ppFurin expression did not significantly alter the plasma membrane expression of Orai1, and similar results were observed with TRPC6, thus suggesting that the mechanism underlying the increase in SOCE detected in ppFurin-expressing MDA-MB-231 cells does not involve enhanced Orai1 surface exposure.

### 2.3. Activation of TRPC6 Channels in MDA-MB-231 ppFurin-Expressing Cells

**Figure 6:**
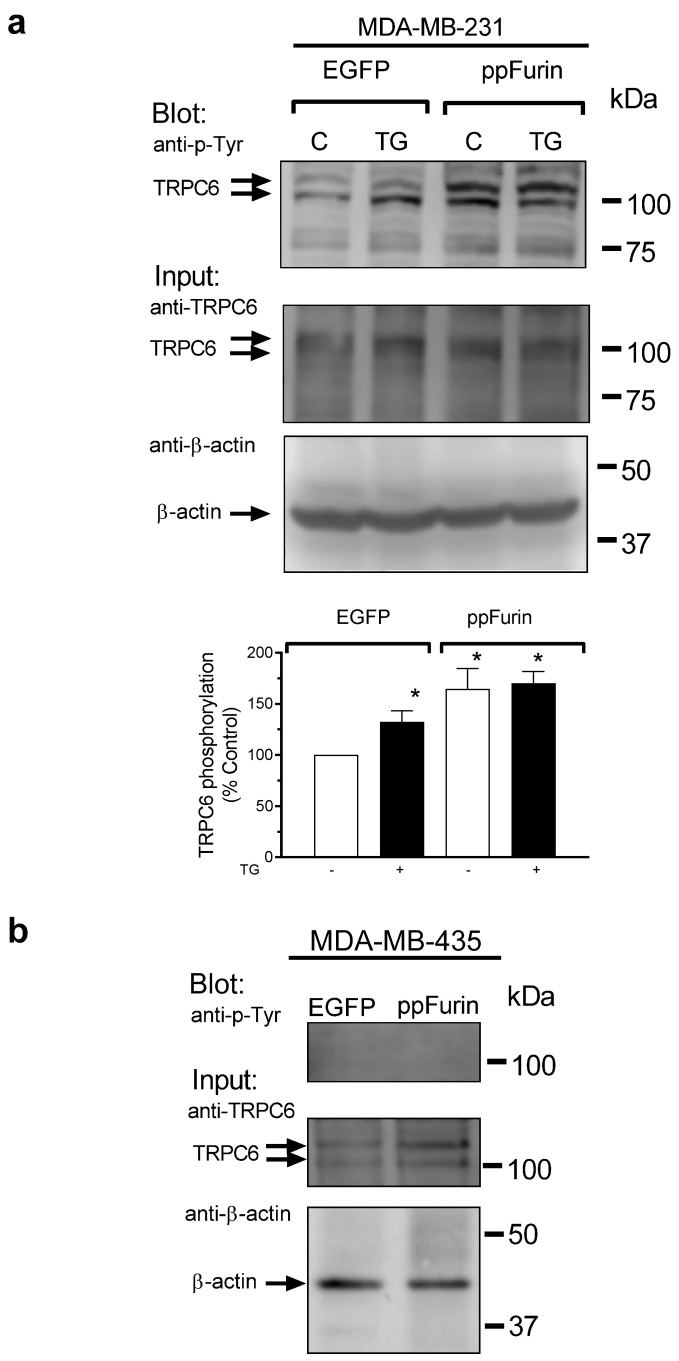
ppFurin induces TRPC6 tyrosine phosphorylation in MDA-MB-231 cells. (**a**) Control and ppFurin-expressing MDA-MB-231 cells were stimulated with 1 µM of TG for 3 min or left untreated and lysed. Whole cell lysates were immunoprecipitated (IP) with anti-TRPC6 antibody. Immunoprecipitates were analyzed by Western blotting using a specific anti-phosphotyrosine antibody and later, protein loading control was carried out by the reprobing of the membrane with the anti-TRPC6 antibody used for immunoprecipitation. Blot images show results representative of five experiments. Bar graphs represent the quantification of TRPC6 tyrosine phosphorylation in resting and TG-treated cells. Results are presented as percentage of control (cells transfected with pIRES2-EGFP empty vector and not stimulated with TG) and expressed as mean ± SEM. Statistical significance was assessed by one-way analysis of variance combined with the Dunnett post hoc test and * represents *p* < 0.05 as compared to control. (**b**) Control and ppFurin-expressing MDA-MB-435 cells were lysed and whole cell lysates were immunoprecipitated (IP) with anti-TRPC6 antibody. Immunoprecipitates were analyzed by Western blotting using a specific anti-phosphotyrosine antibody, and later, protein loading control was carried out by the reprobing of the membrane with the anti-TRPC6 antibody used for immunoprecipitation. Blot images show results representative of five experiments.

Next, we explored the mechanism underlying the activation of TRPC6 by ppFurin. Our results indicate that TRPC6 expression is similar in control and ppFurin-expressing cells (Figure 3 and Figure 4). TRPC6 has been reported to be activated by tyrosine phosphorylation [30]; hence, we explored whether ppFurin is able to induce TRPC6 tyrosine phosphorylation in MDA-MB-231 cells. As depicted in Figure 6, in MDA-MB-231 cells transfected with pIRES2-EGFP empty vector, treatment with TG resulted in a significant increase in the phosphotyrosine level of TRPC6. Interestingly, expression of ppFurin in these cells significantly increased TRPC6 tyrosine phosphorylation, and thus activation, under resting conditions, and TG was unable to induce a further increase in the phosphotyrosine level of the channel (Figure 6a). These findings might explain the constitutive Ca^2+^ entry induced by ppFurin in MDA-MB-231 cells. By contrast, TRPC6 tyrosine phosphorylation was undetectable in MDA-MB-435 cells under resting conditions and ppFurin expression did not induce any detectable increase in the phosphotyrosine content of TRPC6 (Figure 6b). Consistent with the lack of phosphorylation relevant for TRPC6 activation, TRPC6 knockdown did not affect SOCE in MDA-MB-435-eGFP or ppFurin-expressing MDA-MB-435 cells (Figure 5g–j).

### 2.4. Inhibition of MDA-MB-231 Malignant Phenotype by ppFurin

**Figure 7:**
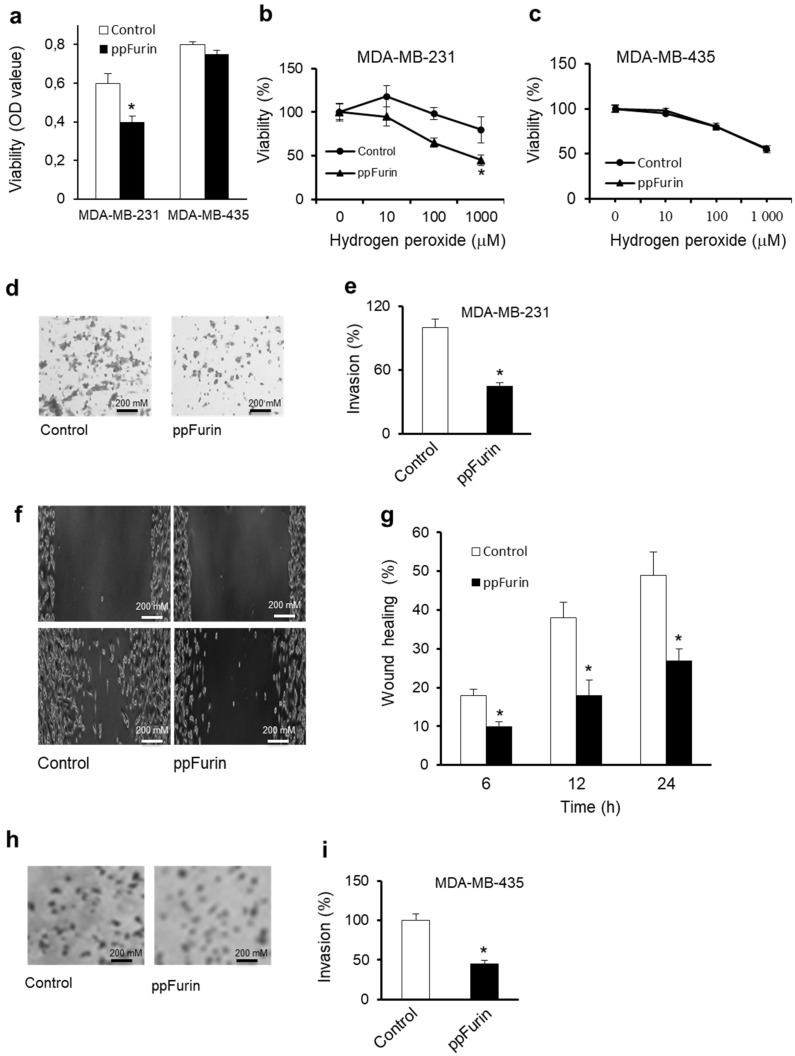
Effect of ppFurin on MDA-MB-231 cells malignant phenotype. (**a**) The optical density (OD) value measured from MTT assay represented basal cell viability of indicated control and ppFurin cells. (**b**,**c**) Effect of hydrogen peroxide on control and ppFurin-expressing MDA-MB-231 (**a**) and MDA-MB-435 (**b**) cells’ viability as assessed by MTT assay and calculated with respect to control, which is set as 100%. (**d**,**e**) Control and ppFurin-expressing MDA-MB-231 cells’ invasion. (**f**,**g**) Cell motility was analyzed by scratch wound assay. (**f**) Control and ppFurin-expressing MDA-MB-231 cells were subjected to scratch wounds and imaged after 24 h (n = 6/well). (**g**) Quantification of wound closure after 6, 12, and 24 h. (**h**,**i**) Control and ppFurin-expressing MDA-MB-435 cells’ invasion. All data are representative of 3 experiments and shown as mean ± S.E.M (n = 3 per group). * *p* < 0.05. Student’s *t*-test was applied for statistical analysis. *p* values of <0.05 were considered significant.

Previously induction of TRPC6 was reported to promote the aggressive phenotype of various cancer cells by promoting a sustained elevation of intracellular Ca^2+^ level, which is critical for cancer cells proliferation and migration [9]. While other studies reported that TRPC6 overactivation mediated a significant reduction in breast cancer cells growth and viability [26]. Thereby, we first determined whether the TRPC6 activation in ppFurin-expressing MDA-MB-231 cells was accompanied by alteration in their viability and resistance to the apoptotic agent hydrogen peroxide (H_2_O_2_). As illustrated in Figure 7a, the expression of ppFurin in these cells affected their basal viability while compared to MDA-MB-435 cells. Cells treatment for 6 h with various concentrations of H_2_O_2_ inhibited MDA-MB-231 cells viability in a dose-dependent manner that was further enhanced in the presence of ppFurin (Figure 7b). In contrast, H_2_O_2_ mediated a similar effect on MDA-MB-435 cells’ viability in the presence and absence of ppFurin (Figure 7c). Using the invasion assay revealed that the expression of ppFurin in MDA-MB-231 repressed their ability to invade collagen (Figure 7d,e). Similarly, the scratch wound healing assay revealed that ppFurin delayed MDA-MB-231 monolayer repair at all the time points analyzed (Figure 7f,g). Expression of ppFurin in MDA-MB-435 cells repressed their invasion as observed for MDA-MB-231-ppFurin-expressing cells (Figure 7h,i).

### 2.5. Effect of ppFurin Expression on Ca^2+^ Influx, Cell Migration, and Viability in Triple Negative BT20 Breast Cancer Cells

**Figure 8:**
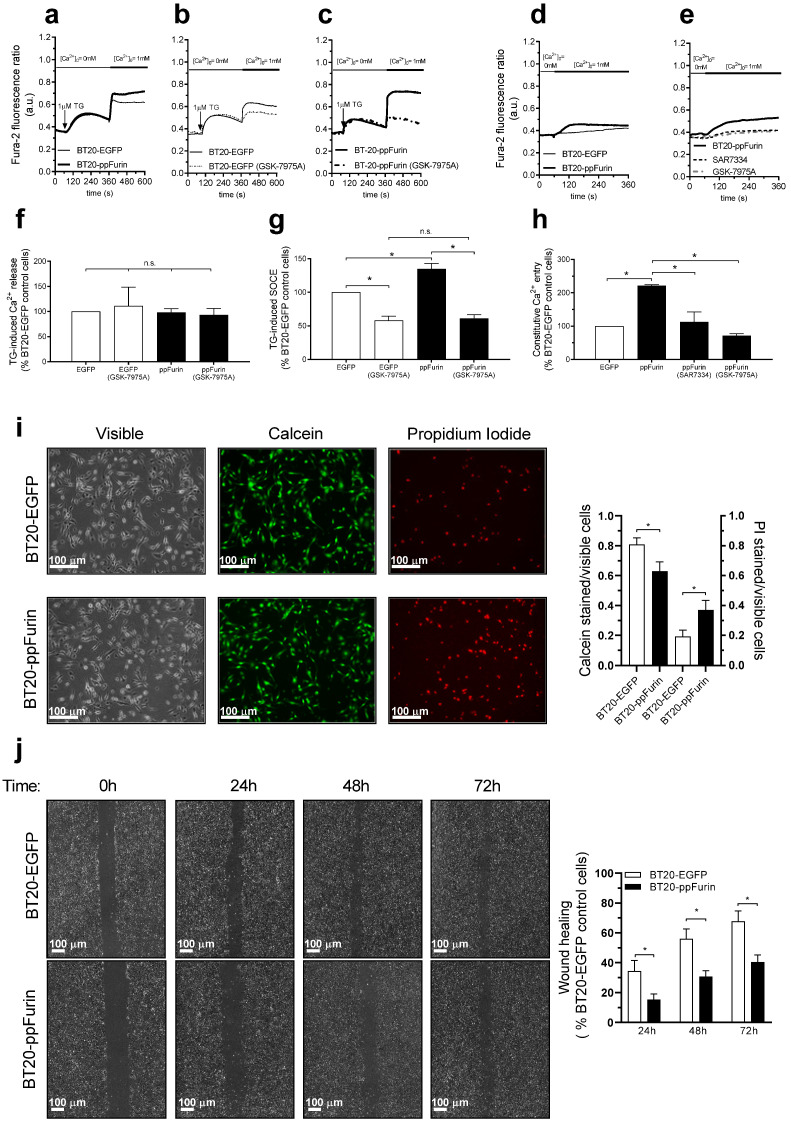
Effect of ppFurin expression on Ca^2+^ entry, cell migration, and viability in triple negative BT20 breast cancer cells. (**a**–**h**) Control BT20 and BT20 cells expressing ppFurin were loaded with Fura-2 and perfused with a Ca^2+^-free medium (100 µM of EGTA added). Cells were pretreated for 1 min with 10 µM of GSK-7975A or the vehicle (**b**,**c**,**e**) or for 10 min with 1 µM of SAR7334 (**e**). Cells were then stimulated with TG (1 µM) followed by reintroduction of external Ca^2+^ (final concentration 1 mM) to initiate Ca^2+^ entry (**a**–**c**), or were subjected to Ca^2+^ re-addition to determine the existence of constitutive Ca^2+^ entry (**d**,**e**). Traces are representative of 40 cells/day/3–5 days. (**f**–**h**) Bar graphs represent TG-induced Ca^2+^ release (**f**), SOCE (**g**), and constitutive Ca^2+^ entry (**h**), as indicated, as the area under the curve, expressed as mean ± SEM, and presented as percentage of control (BT20-EGFP control cells). Statistical significance was assessed by Student’s *t*-test and * represents *p* < 0.05 as compared to their respective controls. (**i**) Control BT20 and BT20 cells expressing ppFurin cells were loaded with calcein and propidium iodide (PI). Cell staining was visualized using an inverted microscope as described in the Material and Methods. Bar graphs represent the calcein and propidium iodide staining under the different conditions expressed as the ratio between stained vs. visible cells and are expressed as mean ± SEM. Images shown are representative of 6 independent experiments. Statistical significance was assessed by Student’s *t*-test and * represents *p* < 0.05 as compared to cells transfected with pIRES2-EGFP empty vector. (**j**) Control BT20 and BT20 cells expressing ppFurin cells were subjected to wound healing assay as described in the Methods. Representative images were acquired at 0, 24, 48, and 72 h from the beginning of the assay. Bar graphs represent the quantification of wound closure after 24, 48, and 72 h expressed as the mean ± SEM of 4 independent experiments. Analysis of statistical significance was performed by Student’s *t*-test and * represents *p* < 0.05 as compared to cells transfected with pIRES2-EGFP empty vector.

We further explored whether the effect of ppFurin expression is limited to MDA-MB-231 cells or it also occurs in other triple negative breast cancer cells. As reported above for MDA-MB-231 cells, expression of ppFurin in BT20 cells results in enhancement of SOCE in response to treatment with TG, without having any effect on TG-induced Ca^2+^ release (Figure 8a,f,g). As for MDA-MB-231 cells (see Figure 5), pretreatment with GSK-7975A significantly attenuated SOCE in these cells, and the remaining SOCE after treatment with the inhibitor was similar in control and ppFurin-expressing BT20 cells (Figure 8b,c,g), which further confirms that the enhanced SOCE observed in ppFurin-expressing cells depends on the function of Orai channels. Furthermore, while control BT20 cells do not exhibit constitutive Ca^2+^ influx, a significant Ca^2+^ influx was detectable in non-stimulated ppFurin-expressing BT20 cells (Figure 8d,e,h). Pretreatment with the Orai channel inhibitor, GSK-7975A, or the TRPC6 inhibitor, SAR7334, significantly reduced constitutive Ca^2+^ entry in these cells. These findings indicate that, as observed in MDA-MB-231 cells, ppFurin-evoked constitutive Ca^2+^ entry involves both Orai and TRPC6 channels. In addition, we have explored the effect of ppFurin expression on cell viability and migration, and we have found that ppFurin expression significantly attenuated BT20 cell viability as detected using calcein/propidium iodide staining (Figure 8i). Finally, the wound healing assay revealed that ppFurin expression significantly attenuated the ability of BT20 cells to migrate (Figure 8j). Therefore, the effect of ppFurin expression occurs, at least, in two unrelated triple negative breast cancer cells.

## 3. Discussion

Proteolytic maturation of protein precursors by furin is involved in the activation of different cellular mechanisms that are directly responsible for the malignant phenotype of a variety of tumors, including breast cancer [10,20,31]. These processes often involve the activation of proteins required for cell proliferation, invasion, and signaling pathways that sustain cancer cell growth and viability [10,20,31]. We have previously reported that inhibition of protein precursors’ cleavage can affect these processes in different tumor cell types [14,15,16,17,19]. In particular, inhibition of the proprotein convertases (PCs) by the exogenous general PC inhibitor α1-PDX elicits the repression of the activity of all the PCs found in the secretory pathway leading to the inhibition of a variety of growth factors, receptors, and proteases, resulting in reduced tumor cells proliferation, invasion, and/or survival types [14,15,16,17,19].

Since Ca^2+^ signaling and various Ca^2+^-regulating proteins known to be involved in breast tumor development and progression are substrates and/or downsteam effectors of the ubiquitously expressed PC furin [15,32], we thought that ppFurin [33], its naturally occurring inhibitor, might be involved in the regulation of Ca^2+^ mobilization in breast cancer cells. By analyzing the effect of stable expression of ppFurin on PCs activity in MDA-MB-231 breast cancer and MDA-MB-435 melanoma cells, we found that ppFurin significantly reduces the in vitro enzymatic activity of the PCs. This was confirmed following the analysis of two distinct furin substrates, proVEGF-C and proIGF-1R, and it was found that ppFurin was driving in the two cell lines the cleavage repression of these protein precursors. Further analysis revealed that expression of ppFurin in triple negative breast cancer MDA-MB-231 and BT20 cells, but not in MDA-MB-435 melanoma cells or in MCF-7 luminal breast cancer cells, significantly induces constitutive Ca^2+^ entry. This Ca^2+^ mobilization was found to imply TRPC6 and Orai channels’ activation, since constitutive Ca^2+^ entry was disturbed following silencing of TRPC6 expression, as assessed by means of the shRNA gene knockdown approach, and by pharmacological inhibition of TRPC6 and Orai channels with SAR7334 or GSK-7975A, respectively. Furthermore, our results indicate that ppFurin expression leads to enhanced SOCE in MDA-MB-231 and BT20, but not in MDA-MB-435 cells, an effect that was strongly dependent on Orai1 activity. We have recently reported that in MDA-MB-231 cells, cation influx through TRPC6 is required for plasma membrane expression of Orai1 [9], which is the main channel for SOCE in these cells [8]. However, we found that while ppFurin expression in MDA-MB-231 is associated with an increase in TRPC6 tyrosine phosphorylation and activation, this event is not accompanied by an increase in Orai1 surface expression. These findings indicate that SOCE might be enhanced by activation of Orai1 while non-capacitative Ca^2+^ entry is induced by activation of TRPC6 and Orai channels in ppFurin-expressing cells.

Targeting altered Ca^2+^ homeostasis in cancer cells was previously proposed as a potential anti-cancer strategy by interfering with the expression and/or the activity of Ca^2+^-handling proteins. Indeed, the activity of various Ca^2+^ channels is known to be important in the control of cancer cells growth and survival [7,32]. Specifically, Orai1 and TRPC6 channels are well established to be implicated in cell proliferation and migration [9] and various hypertrophic gene expression is reported to be mediated by NFAT pathway activation in normal [34] and cancer [35] cells. In this aspect, Orai1, complexed with the SK3 channel, has been found to play a relevant role in breast cancer cell migration [36] and inhibition of TRPC6/Orai1-mediated SOCE was found to attenuate breast cancer cell proliferation and migration [9]. In the current studies, we found that Orai1 and TRPC6 function was upregulated in MDA-MB-231 ppFurin-expressing cells compared to control MDA-MB-231 cells, leading to increased Ca^2+^ entry. The relevance of Orai1 in breast cancer cell function has been widely demonstrated. A recent study has revealed the role of Orai1, together with the channel Kv10.1 and the secretory pathway Ca^2+^-ATPase-2 (SPCA2), in collagen-induced breast cancer cell survival ([37]). Furthermore, Orai1-mediated SOCE has been found to be required for cell migration. In breast and lung cancer cells, soluble αKlotho has been reported to attenuate SOCE by inhibiting PI3K-driven cell surface expression of Orai1, leading to the suppression of SOCE-mediated tumor cell migration [38]. Agonist-evoked Ca^2+^ signals through the activation of Orai1 also fine tune breast cancer cell functions as described in triple negative breast cancer cells where progesterone attenuates cell proliferation by a mechanism involving Ca^2+^ entry mediated by STIM2, Orai1, and TRPC1 [39]. These findings suggest that Orai1-mediated SOCE is essential to fine tune different breast cancer hallmarks. Moreover, the role of TRPC6 hyperactivity in the inhibition of cell growth and induction of apoptosis has been previously demonstrated [26]. Indeed, while TRPC6 activation is known to promote cell proliferation, its overactivation by hyperforin was reported to induce a significant disorder in Ca^2+^ signaling that affects cell proliferation and induces apoptosis in cancer cells [26,40]. Similarly, extended enhancement in [Ca^2+^]_i_ was reported to activate various apoptotic cascades that encouraged the use of drugs able to maintain enhanced [Ca^2+^]_i_ in cancer treatment. These include topotecan [41], arsenic trioxide (As_2_O_3_) [42], and 5-Fluorouracil (5-FU) [43], all inducing intracellular Ca^2+^ alteration that leads to apoptosis. For example, 5-FU, which is used for the treatment of various cancers, including breast cancer, involves the elevation of [Ca^2+^]_i_ that activates p53, resulting in the activation of caspases and lately apoptosis [43,44]. In addition to cell growth and proliferation, Ca^2+^ signaling also plays a central regulatory role in migration [45]. We found that ppFurin expression in MDA-MB-231 cells leads to hyperactivation of Ca^2+^ influx through Orai and TRPC6 channels as well as reduction in cell migration and invasion, suggesting that Ca^2+^ entry through hyperactive TRPC6 and Orai channels may contribute to the migration and invasion abilities of MDA-MB-231 cells but require further investigations. Although how TRPC6 hyperactivation affects MDA-MB 231 cells motility is quite unclear, the role of TRPC6 in the regulation of the actin cytoskeleton system activity may be the major contributing factor. Indeed, TRPC6-mediated Ca^2+^ influx was reported to be directly associated with actin cytoskeleton that is responsible for the regulation of cell morphology, cell adhesion, and motility [45]. While the TRPC6-mediated Ca^2+^ entry was found to contribute to migration and invasion, the gain-of-function mutation of TRPC6 found in several podocytes leads to focal and segmental glomerulosclerosis (FSGS), a leading cause of glomerulonephritis and chronic kidney disease [46,47] and proteinuria [48] due to alteration of actin organization and subsequently cell migration repression. In addition, exaggerated Ca^2+^ signaling mediated by these TRPC6 mutations was also found to cause apoptosis [47]. Accordignly, TRPC6 inhibition or knockout was reported to have a protective effect on H_2_O_2_-induced apoptosis, whereas TRPC6 expression both triggers Ca^2+^ influx through SOCE and make cells more vulnerable to death [49]. In our model, the ability of ppFurin to induce TRPC6 activation may explain at least in part the reduced viability found in MDA-MB-231 cells stably expressing ppFurin. Interestingly, while ppFurin expression in MDA-MB-231, BT20, and MDA-MB-435 cells repressed their ability to migrate or invade, ppFurin affected viability only in the two breast cancer cell lines where Orai and TRPC6 hyperactivation was observed. However, it is not ruled out that the effect of ppFurin on MDA-MB-435 cells’ viability cannot be detected due to their high sensitivity to H2O2 that prevent the observation of additional effect of ppFurin. In addition, the inability of the calcium machinery of MDA-MB-435 cells to respond to ppFurin may be linked to their level of PC expression and/or activity. ppFurin was previously reported to repress migration and invasion in various cancer cells including colon cancer cells [14,50] and head and neck squamous cell carcinoma cell lines through inhibition of MMPs activity [51].

An increasing number of studies have identified a wide range of protein precursors in breast cancer cells of which the cleavage and activity are inhibited by furin repression [10,17,31,33,52]. These studies highlight the important role for the cleavage of these protein precursors in breast cancer development and progression and reveal that their cleavage inhibition results in tumor cells proliferation, invasion, and survival inhibition. However, further investigations are needed to explore the effect of specific protein precursor cleavage inhibition on Orai1- and TRPC6-mediated Ca^2+^ entry. Similarly mechanisms that mediate Orai channels’ activation in the presence of ppFurin expression remain to be investigated.

## 4. Materials and Methods

### 4.1. Materials and Reagents

Fura-2 acetoxymethyl ester (fura-2/AM) was from Molecular Probes (Leiden, The Netherlands). Thapsigargin (TG), rabbit polyclonal anti-Orai1 antibody (catalog number O8264, epitope: Amino acids 288–301 of human Orai1), mouse monoclonal anti-Orai3 antibody (clone 1B4F1, epitope: 19 amino acid peptide from near the C-terminus), rabbit polyclonal anti-β-actin antibody (catalog number A2066, epitope: Amino acids 365–375 of human β-actin), and bovine serum albumin (BSA) were from Sigma (Madrid, Spain). Mouse monoclonal anti-STIM1 antibody (Clone 44/GOK, epitope: Amino acids 25–139 of human STIM1, catalog number 610954) was from BD Transduction Laboratories (Franklin Lakes, NJ, USA). Rabbit polyclonal anti-STIM2 antibody (catalog number PA5-20372) was purchased from Invitrogen (Madrid, Spain). Rabbit polyclonal anti-TRPC6 antibody (catalog number: TA328771, corresponding to amino acid residues 573–586 of rat TRPC6, seconnd extracellular loop) was from Origene (Rockville, MD, USA). The anti-insulin-like growth factor I receptor (IGF-1R) and anti-vascular endothelial growth factor C (VEGF-C) were from Santa Cruz Biotechnology (Dallas, TX, USA) Mouse-monoclonal anti-phosphotyrosine antibody (catalog number: 05-321) was from Millipore (Temecula, CA, USA). Rabbit polyclonal anti-TRPC1 antibody (catalog number PA5-77303, epitope: Peptide corresponding to amino acid residues 557–571) and Live/Dead^®^ viability/cytotoxicity kit were from Thermo Fisher (Madrid, Spain). All other reagents were of an analytical grade.

### 4.2. Cell Culture and Transfections

The characteristics and the origin of the control and stably ppFurin-transfected human breast cancer MDA-MB-231 and the melanoma MDA-MB-435 cell lines were described previously [50]. Using the same procedure, the breast cancer cells BT20 and MCF7 were also transfected with empty vector or vector expressing ppFurin. In other experiments, MDA-MB-231 and MDA-MB-435 cell lines were transiently transfected with pcDNA3-zeo-Flag.cm5 empty vector or the same vector containing wild-type VEGF-C. Transfections were carried out using the Effectene transfection reagent (Qiagen, Hilden, Germany) in accordance with the manufacturer’s instructions and stably ppFurin-expressing cells were selected using G418 resistance, as previously described [53,54]. Cells were grown in DMEM supplemented with 10% FCS, 100 units/mL of penicillin, and 100 mg/mL of streptomycin.

### 4.3. RNA Extraction and Real Time PCR

Total RNA extraction was performed using the Macherey–Nagel RNA isolation kit that includes DNase treatment (Qiagen, Hilden, Germany), in accordance with the manufacturer’s instructions. The RNA was reverse transcribed using the high-capacity cDNA reverse transcription kit (Applied Biosystems, Courtaboeuf, France) and used for real time PCR in the presence of specific primers and Power SYBR Green PCR Master Mix (Applied Biosystems, Courtaboeuf, France), as previously described [12,14]. The quantitative polymerase chain reaction (qPCR) data were acquired with the StepOnePlusTM Real-Time PCR System (Applied Biosystems, Courtaboeuf, France). The expression levels were normalized to human GAPDH.

### 4.4. Immunoprecipitation and Western Blotting

Immunoprecipitation and Western blotting were performed as described previously [55]. Briefly, 500-µL aliquots of cell suspension (18 × 106 cell/mL) were lysed with an equal volume of ice-cold 2 × NP-40 buffer, containing 274 mM of NaCl, 40 mM of Tris, 4 mM of EDTA, 20% glycerol, 2% nonidet P-40, 2 mM of Na_3_VO_4_, and complete EDTA-free protease inhibitor tablets. Samples of cell lysates (1 mL) were immunoprecipitated by incubation with 2.5 µg of anti-TRPC6 and 50 µL of protein A-agarose overnight at 4 °C on a rotary platform. The immunoprecipitates were resolved by 10% SDS-PAGE and separated proteins were electrophoretically transferred onto nitrocellulose membranes for subsequent probing. Blots were incubated for 3 h at room temperature with 10% (w/v) BSA in tris-buffered saline with 0.1% Tween 20 (TBST) to block residual protein binding sites. Immunodetection of phosphotyrosine, Orai1, Orai3, STIM1, STIM2, TRPC1, TRPC6, and PMCA was achieved by incubation for 1 h with anti-Orai1 antibody diluted 1:1000 in TBST, 1 h with anti-STIM2 antibody diluted 1:500 in TBST or overnight with anti-phosphotyrosine, anti-Orai3, anti-STIM1, anti-TRPC1, anti-TRPC6, or anti-PMCA antibody diluted in 1:500 in TBST. The primary antibody was removed, and blots were washed three times for 10 min each with TBST. Primary antibodies were detected by incubation for 1 h with horseradish peroxidase-conjugated goat anti-mouse IgG antibody or horseradish peroxidase-conjugated goat anti-rabbit IgG antibody diluted 1:10000 in TBST and then exposed to enhanced chemiluminescence reagents for 5 min. IGF-1R and VEGF-C cleavage detection by Western blotting analysis was performed, as previously described [14,22]. The density of bands was measured using C-DiGit Chemiluminescent Western Blot Scanner (LI-COR Biosciences, Lincoln, NE, USA) and ImageJ software v.1.8.0_172 (NIH, Bethesda, MD, USA) Data were normalized to the amount of protein recovered by the antibody used for the immunoprecipitation (Figure S1).

### 4.5. Determination of Cytosolic Free-Ca^2+^ Concentration

Cells were loaded with Fura-2 by incubation with 2 μM of Fura-2/AM for 30 min at 37 °C. Coverslips with cultured cells were mounted on a perfusion chamber and placed on the stage of an epifluorescence inverted microscope (Nikon Eclipse Ti2, Amsterdam, The Netherlands) with image acquisition and analysis software for videomicroscopy (NIS-Elements Imaging Softwarev.5.02.00, Nikon, Tokyo, Japan).. Cells were superfused with HEPES-buffered saline (HBS) containing (in mM): 125 of NaCl, 5 of KCl, 1 of MgCl_2_, 5 of glucose, 25 of HEPES, and pH 7.4, supplemented with 0.1% (w/v) BSA, and were alternatively excited with light from a xenon lamp passed through a high-speed monochromator (Optoscan ELE 450, Cairn Research, Faversham, UK) at 340/380 nm. Fluorescence emission at 505 nm was detected using a sCMOS camera (Zyla 4.2, Andor, Belfast, UK) and recorded using NIS-Elements AR software (Nikon, Amsterdam, The Netherlands). The fluorescence ratio (F340/F380) was calculated pixel by pixel [56]. TG-evoked Ca^2+^ release and SOCE were measured as the integral of the rise in the fura-2 fluorescence ratio for 2½ min after the addition of TG or Ca^2+^, respectively, taking a sample every second.

### 4.6. Cell Viability Assay

The cell viability was evaluated using the MTT assay, as previously described [57]. Control and ppFurin-expressing cells were plated in 96-well microtiter plates, respectively, at a density of (1 × 10^4^/^mL^) cells/well. The next day, the basal viability of MDA-MB-231 and MDA-MB-435 was determined and cells were treated with various concentrations of H_2_O_2_ for 6 h and were immersed with an MTT solution (0.5 mg/mL) for 3 h at 37 °C. Then absorbance was measured at 570 nm using the ELISA plate reader. Alternatively, cell viability was tested using the Live/Dead^®^ viability/cytotoxicity kit as described previously [9]. Briefly, cells were incubated with calcein-AM and propidium iodide following the manufacturer’s instructions, and samples were excited at 430 and 555 nm for calcein and propidium iodide, respectively. Fluorescence emission at 542 (for viable cells) and 624 nm (for dead cells) was recorded using an epifluorescence inverted microscope (Nikon Eclipse Ti-2).

### 4.7. Cell Invasion Assay

The in vitro invasion activity of control and MDA-MB-231 ppFurin-expressing cells was performed using 24-well microchemotaxis chambers precoated with 7.5 μg of collagen type IV (Becton Dickinson Labware, Franklin Lake, NJ, USA), as previously described [22,31]. Tumor cells (1 × 10^5^/^mL^) were resuspended in serum-free media and were loaded into the upper chamber of Transwells. Cells were incubated at 37 °C for 24 h, and invasion across collagen layer was initiated by adding 10% FBS (chemoattractant) to the lower chamber. Cells detected in each well were counted and the results were represented as (number of migrated cells/number of total cells) × 100%.

### 4.8. Wound-Healing Assay

The wound-healing assay was conducted as previously reported [52]. Briefly, control and ppFurin-expressing cells were seeded into 6-well plates at 5 × 105/well and grown in growth media until 90–100% confluency. A cell-free area (wound) within the cell monolayer was constructed using a sterile micropipette tip. Healing of the wound was observed at different time periods by a light microscopy and analyzed using Image J software. The area that remained clear of cells after this period was quantified and compared with the area of the wound at time 0.

### 4.9. Biotinylation Protocol

Cells were washed 3 times with phosphate-buffered saline (PBS, NaCl 137 mM, KCl 2.7 mM, KH_2_PO_4_, 1.5 mM, Na_2_HPO_4_•2H_2_O 8mM, pH 7.4), resuspended in biotinylation buffer (50 mM NaHCO_3_ and 0.9% NaCl) and labeled with 100 mg/mL sulfo-NHS-LC biotin at RT. Surface labeling was stopped 1 h later with 1% NH_4_Cl and PBS supplemented with 50 mM of EDTA, and samples were washed twice in PBS/EDTA. Biotinylated cells were lysed with Nonidet P40 buffer (NP40), and lysates were incubated with streptavidin-conjugated agarose beads overnight at 4 °C on a rocking platform. Biotinylated proteins were isolated by centrifugation and washed 3 times in NP40 buffer. The biotinylated and non-biotinylated fractions were separated in 10% SDS-PAGE and analyzed by Western blotting using the anti-PMCA antibody as the control, or either the anti-Orai1 or anti-TRPC6 antibody.

### 4.10. Statistical Analysis

All data are presented as the mean ± standard error of mean (SEM) unless specifically mentioned. Student’s *t*-test or one-way analysis of variance combined with the Tukey post hoc test were applied for statistical analysis, as appropriate. *p*-values of <0.05 were considered significant.

## 5. Conclusions

Our work shows that expression of ppFurin, a naturally occurring inhibitor of the convertase Furin, mediates activation of Orai and TRPC6 that induces enhanced Ca^2+^ influx in triple negative breast cancer cells, which might be associated with the reduced viability and migration and their enhanced sensitization to the apoptotic agent hydrogen peroxide. The mechanism underlying hyperactivation of Orai and TRPC6 channels in triple negative breast cancer cells deserves further investigation. Thus, interfering with Furin activity-mediated Ca^2+^ mobilization and Orai and TRPC6 activation represent a potential strategy controlling malignant phenotype and resistance to therapy in triple negative breast cancer cells. Prospect studies based on our conclusion will validate the efficacy of overactivation of Orai and TRPC6 in breast cancer repression using in vivo models.

## Data Availability

The data presented in this study are available on request from the corresponding author.

## Supplementary Materials

The following are available online at https://www.mdpi.com/article/10.3390/cancers13071670/s1, Figure S1: Uncropped Western Blot Images.
