# Supplementary material for: Furin Prodomain ppFurin Enhances Ca2+ Entry Through Orai and TRPC6 Channels’ Activation in Breast Cancer Cells
Source: Cancers (Basel). 2021 Apr 1;13(7):1670. doi: 10.3390/cancers13071670 (PMC8037623; doi:10.3390/cancers13071670)
Supplement: Supplementary file 1 [file cancers-13-01670-s001.pdf]

Figure S1. Uncropped Western Blot Images

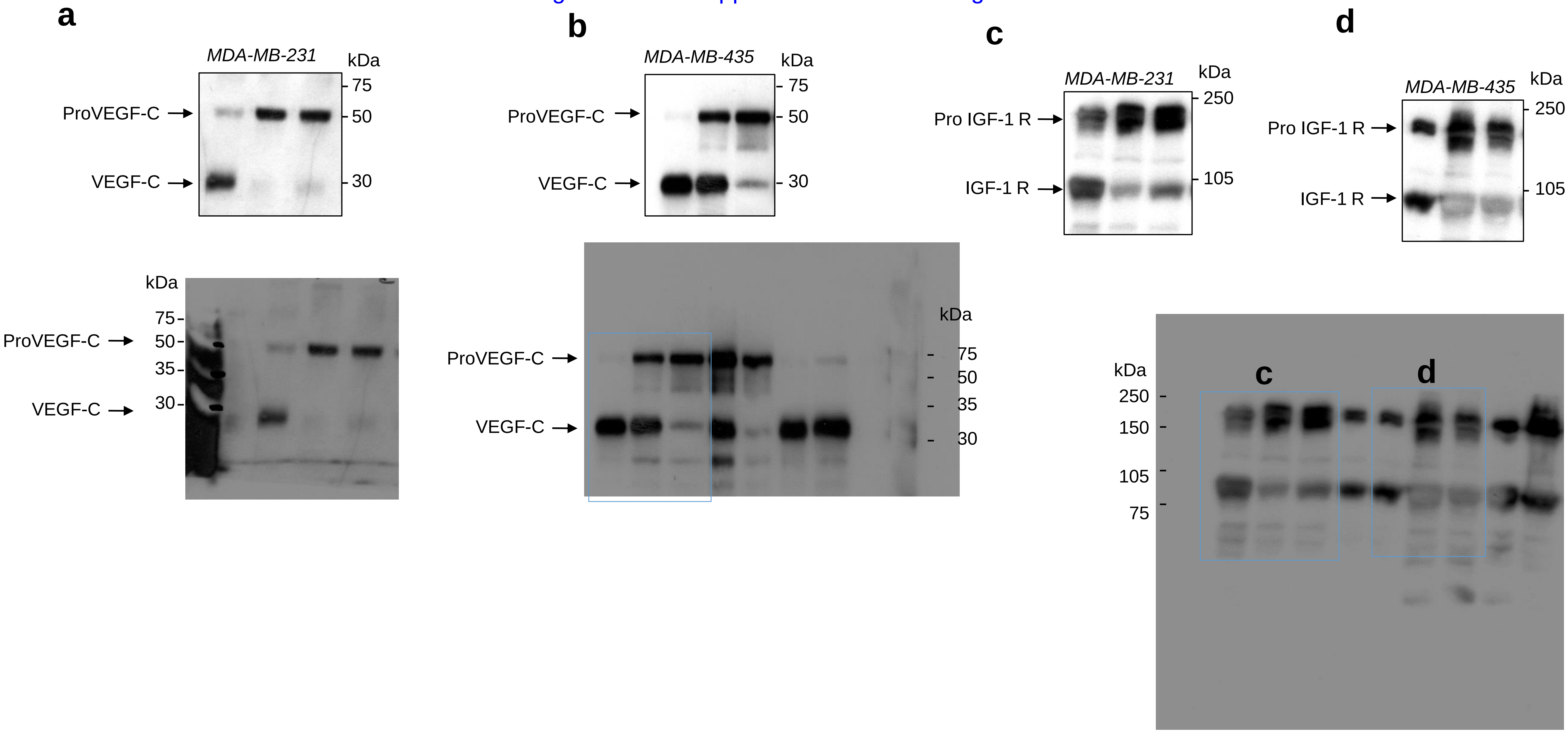

"Uncropped Western Blot images of Fig 2 "

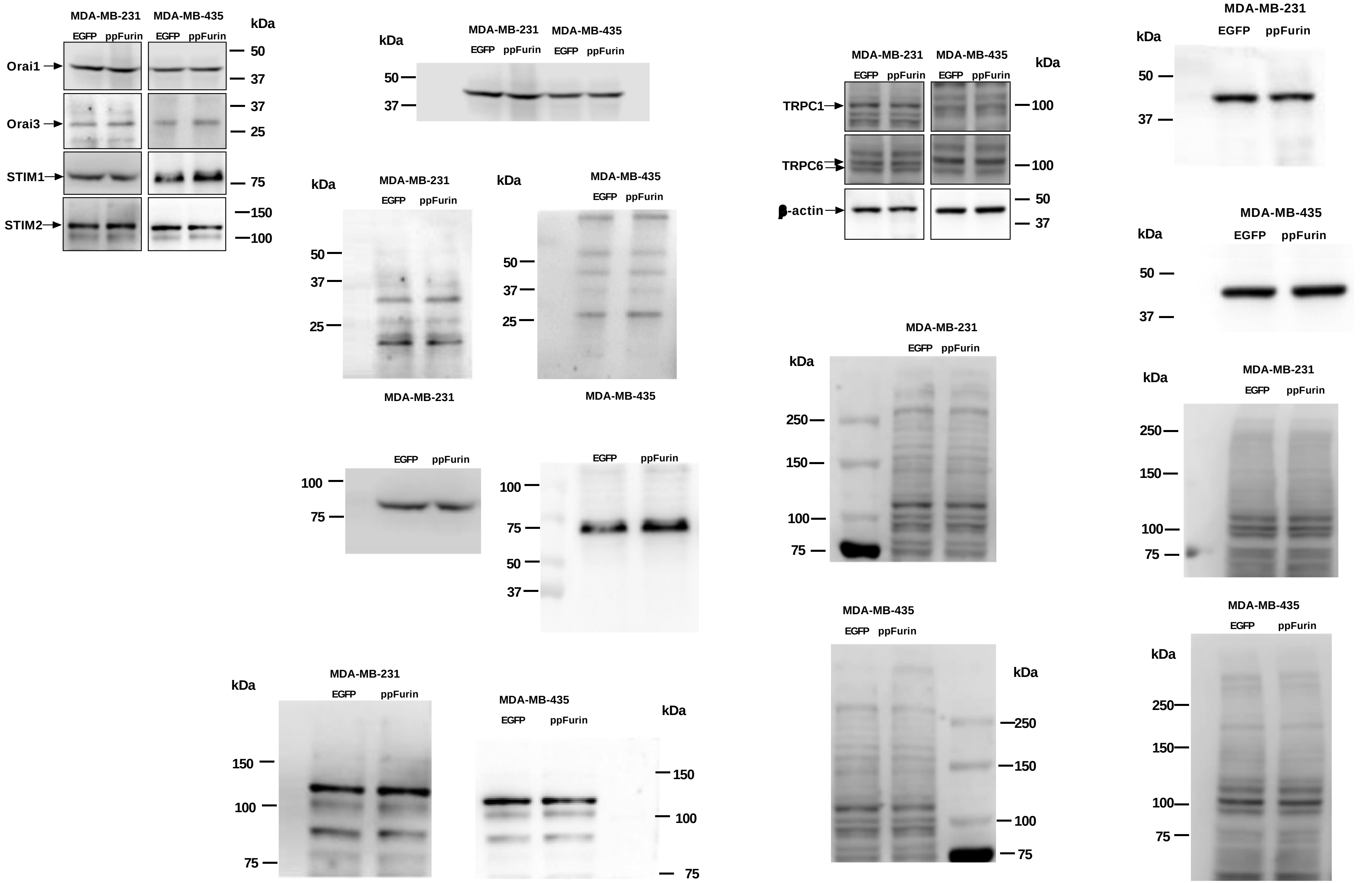

"Uncropped Western Blot images of Fig 3a "

g

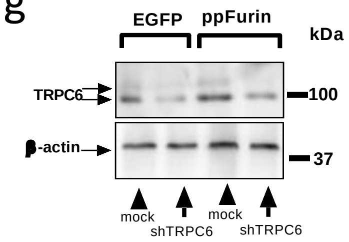

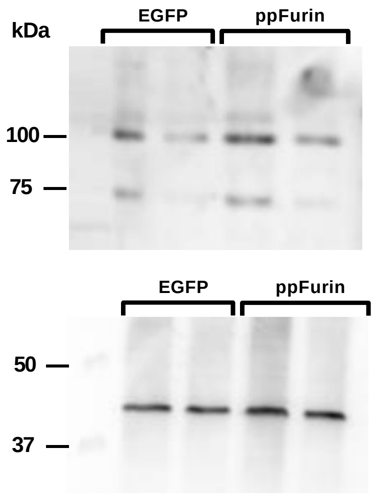

m

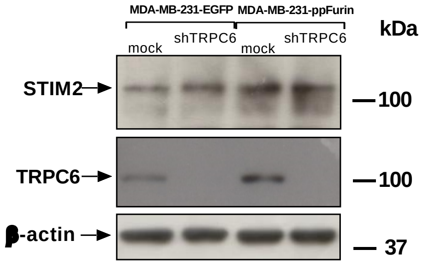

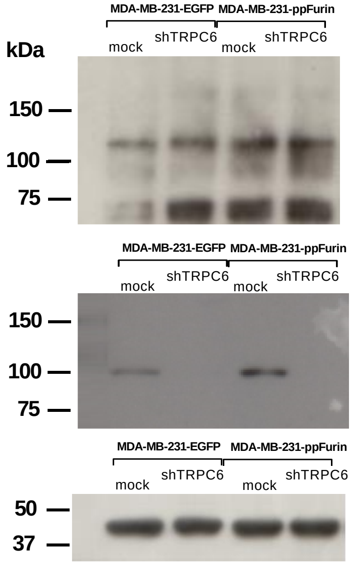

"Uncropped Western Blot images of Fig 4

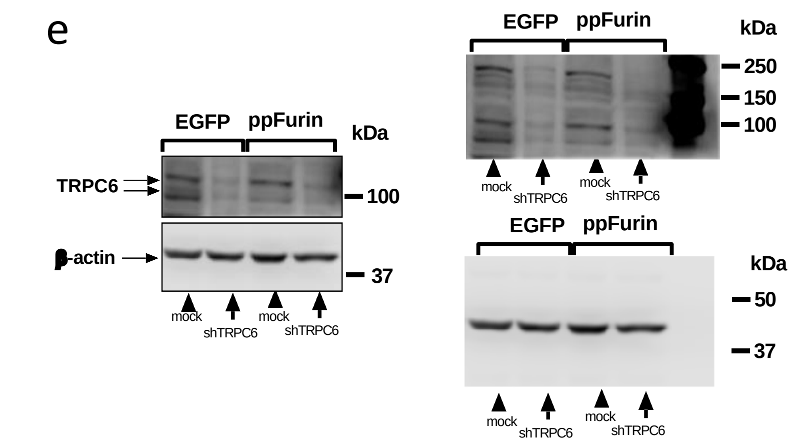

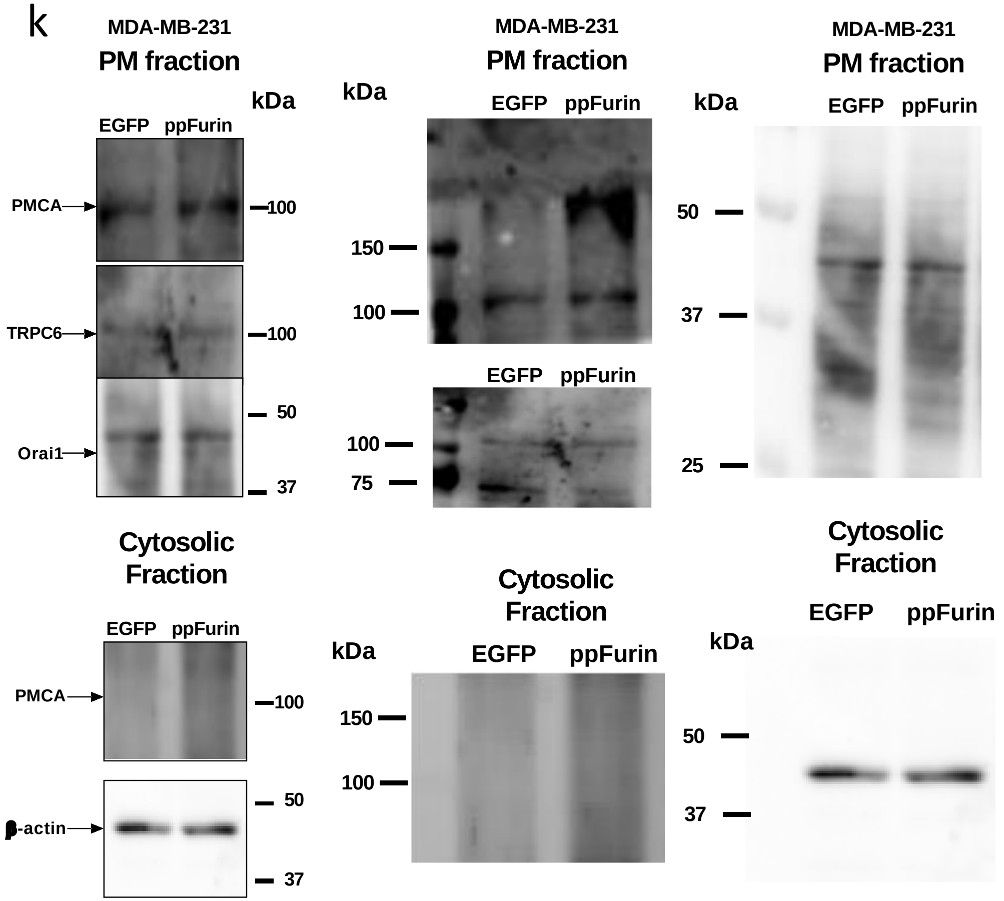

"Uncropped Western Blot images of Fig 5 "

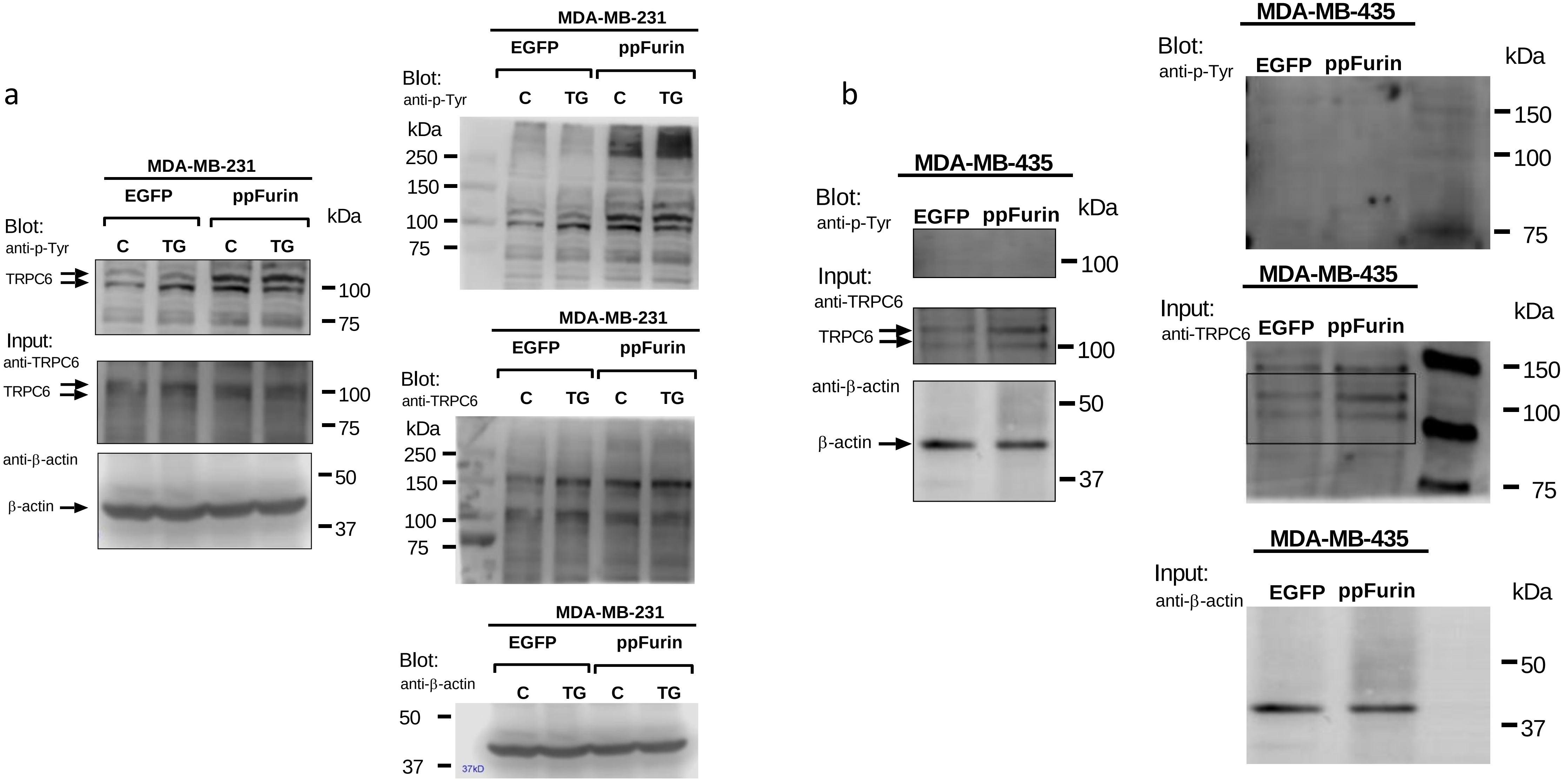

"Uncropped Western Blot images of Fig 6
